# Essential Oil of *Lippia origanoides* Kunth: Nanoformulation, Anticholinesterase Activity, and Molecular Docking

**DOI:** 10.3390/molecules30071554

**Published:** 2025-03-31

**Authors:** Antônio Quaresma da Silva Júnior, Gabriela dos Santos Rodrigues, Adenilson de Sousa Barroso, Pablo Luis Baia Figueiredo, Francisco Paiva Machado, Mikaela Amaral Ferreira, Caio Pinho Fernandes, Gabriela B. dos Santos, Rosa Helena V. Mourão

**Affiliations:** 1 Programa de Pós-Graduação em Biodiversidade e Biotecnologia da Rede Bionorte, Universidade Federal do Oeste do Pará, Santarém 68040-255, PA, Brazil; pablo.figueiredo@uepa.br; 2 Laboratório de Bioprospecção e Biologia Experimental, Universidade Federal do Oeste do Pará, Santarém 68040-255, PA, Brazil; gabrielastsrodrigues@gmail.com (G.d.S.R.); adenilson.barroso@ufopa.edu.br (A.d.S.B.); 3 Programa de Pós-Graduação em Ciências da Saúde, Universidade Federal do Oeste do Pará, Santarém 68035-110, PA, Brazil; gabriela.bds@ufopa.edu.br; 4 Laboratório de Química dos Produtos Naturais, Universidade do Estado do Pará, Belém 66095-015, PA, Brazil; 5 Laboratório de Tecnologia de Produtos Naturais—LTPN, Departamento de Tecnologia Farmacêutica, Faculdade de Farmácia, Universidade Federal Fluminense, Niterói 24241-000, RJ, Brazil; fmachado@id.uff.br (F.P.M.); mikaelaferreira@id.uff.br (M.A.F.); 6 Programa de Pós-Graduação em Ciências Aplicadas a Produtos para a Saúde, Universidade Federal Fluminense, Niterói 24241-000, RJ, Brazil; 7 Laboratory of Phytopharmaceutical Nanobiotechnology, Department of Biological and Health Sciences, Federal University of Amapá, Macapá 68902-280, AP, Brazil; caiofernandes@unifap.br

**Keywords:** carvacrol, acetylcholinesterase nanoemulsion

## Abstract

This study investigates the therapeutic potential of *Lippia origanoides* essential oil (LOEO) in neurological and pharmaceutical applications. The chemical composition of LOEO was analyzed using gas chromatography–mass spectrometry (GC-MS), revealing major constituents, such as carvacrol, thymol, and γ-gurjunene, known for their antioxidant and antimicrobial properties. LOEO demonstrated significant acetylcholinesterase (AChE)-inhibitory activity, particularly in a nanoformulation that enhances bioavailability and stability. Additionally, the major constituent carvacrol, when tested in isolation, also exhibited AChE-inhibitory activity comparable to that of the nanoformulation. Molecular docking analysis indicated strong binding affinities between LOEO compounds and AChE, supporting its therapeutic potential for neurodegenerative diseases like Alzheimer’s. Additionally, in silico pharmacokinetic predictions revealed favorable absorption and blood–brain barrier penetration profiles for key constituents. Despite promising results, this study acknowledges the need for in vivo validation and long-term stability assessments of the nanoformulation. Future research should focus on pharmacodynamic studies and evaluating the oil’s effectiveness in animal models. These findings highlight LOEO as a valuable candidate for developing natural therapies for neurodegenerative diseases.

## 1. Introduction

*Lippia origanoides* Kunth, popularly known as wild oregano and Marajó sage, belongs to the Verbenaceae family and is widely distributed in tropical and subtropical regions of Latin America. This aromatic plant is valued for its medicinal properties, use as a condiment, and production of essential oils [1,2]. In traditional communities, *L. origanoides* has been used in infusions, decoctions, and compresses to treat various conditions, such as respiratory infections, gastrointestinal problems, and inflammatory diseases [3]. Within the scope of Medicinal Plants and Phytotherapeutics in the SUS (Sistema Único de Saúde), the List of Medicinal Plants of Interest to SUS (ReniSUS) was created in 2009, comprising 71 species with therapeutic potential [4]. Thus, *L. origanoides* is a heterotypic synonym of *Lippia sidoides* Cham [5].

The essential oil of *Lippia origanoides* (LOEO) typically presents carvacrol as its main component, which is responsible for many of the bioactive properties attributed to this plant [6,7]. Among these, antimicrobial, anti-inflammatory, and antioxidant activities stand out [8]. Specifically, the antioxidant activity of the LOEO has been associated with its ability to neutralize reactive oxygen species (ROS), reducing oxidative stress and protecting cells from damage. This antioxidant action may have direct implications for the prevention of neurodegenerative diseases [9], such as Alzheimer’s disease (AD) [10].

The correlation between antioxidant and anticholinesterase activity has drawn increasing attention in scientific research. Studies indicate that compounds with antioxidant properties can inhibit the enzyme acetylcholinesterase (AChE) activity, which plays a critical role in the degradation of the neurotransmitter acetylcholine [11]. Reducing AChE activity results in higher acetylcholine levels in the central nervous system (CNS), which is essential for managing pathologies such as AD [12]. Therefore, exploring the anticholinesterase effects of essential oils and their bioactive constituents represents a promising approach to developing innovative therapies.

AD is one of the most prevalent neurodegenerative diseases and represents a significant public health challenge due to the lack of a cure and the limited treatment options available. Currently available drugs, such as AChE inhibitors, delay disease progression but show limited efficacy [13]. Among natural compounds with therapeutic potential, carvacrol has stood out for its ability to inhibit AChE activity and exert neuroprotective effects [14].

Although the LOEO and its main chemical constituents have demonstrated various biological activities, there are still gaps in the literature regarding its potential anticholinesterase activity and its application in nanostructured systems. Incorporating essential oils into nanoformulations, such as nanoemulsions, can enhance their stability, bioavailability, and efficacy while opening new perspectives for developing sustainable and effective pharmaceutical products. They are kinetic stable colloids often constituted by fine droplets of an oil dispersed in an external phase [15].

Despite the promising biological activities of LOEO, its practical application faces challenges related to volatility, low water solubility, and potential degradation under environmental conditions [15]. Nanoformulations, particularly nanoemulsions, offer an effective strategy for overcoming these limitations by improving the stability, bioavailability, and controlled release of bioactive compounds. These systems enhance the solubility of hydrophobic molecules, protect them from degradation, and promote better absorption, which is especially relevant for neurological applications where effective blood–brain barrier penetration is required. Thus, incorporating LOEO into a nanoemulsion could optimize its therapeutic potential, making it a more viable candidate for neurodegenerative disease treatment.

In this context, the present study aimed to evaluate the anticholinesterase activity of the LOEO, the isolated constituent carvacrol, and a nanoformulation developed from this essential oil. Additionally, molecular modeling analyses were performed to investigate the interactions between the oil’s main compounds and the AChE enzyme, and in silico pharmacokinetic studies were performed to assess the feasibility of oral absorption and potential CNS penetration.

## 2. Results

### 2.1. Volatile Constituents of Essential Oils

The essential oil (EO) from *L. origanoides* leaves exhibited an average yield of 3.3%, and its constituents are presented in Table 1. A total of 95% of the constituents were identified, with emphasis on the following major compounds: *p*-cymene (11.10%), thymol (10.20%), carvacrol (41.20%), and γ-gurjunene (11.80%). The chromatogram of the analysis is available as Supplementary Materials, with the major compounds clearly indicated.

### 2.2. Preparation, Characterization, and Thermal Stress of the Lippia origanoides Formulation

**Figure 1:**
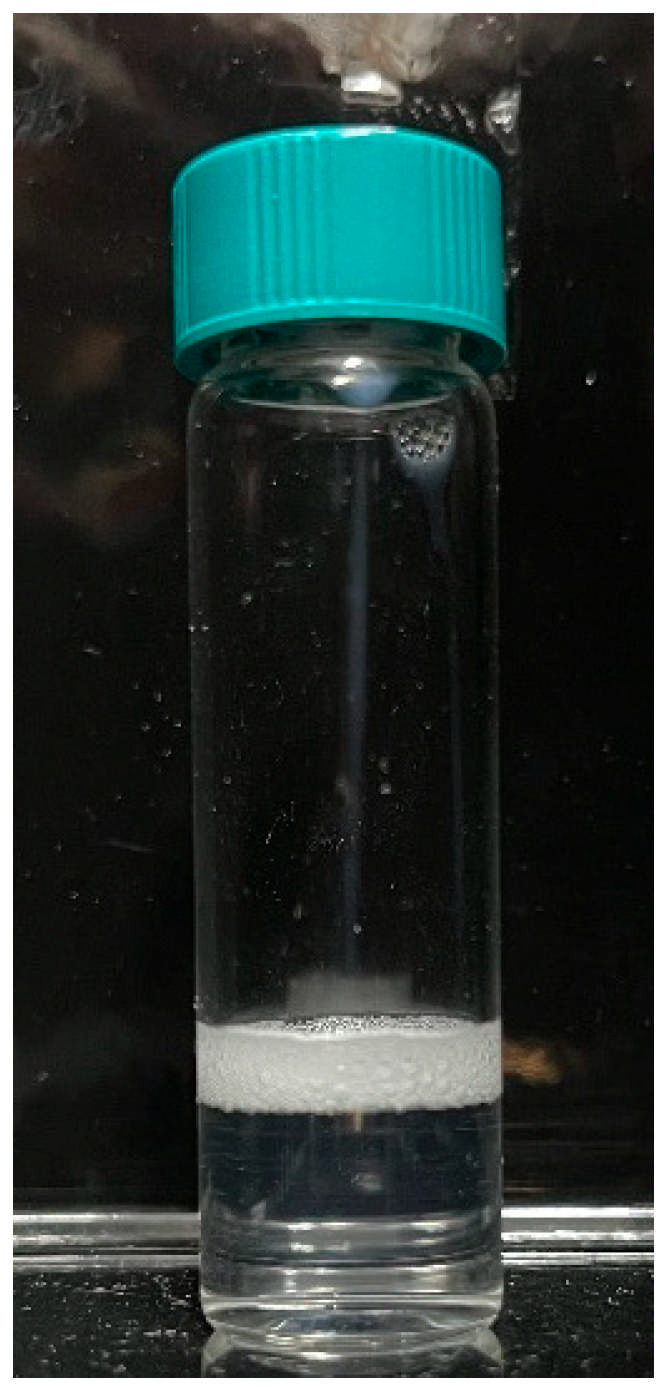
Macroscopic characteristics (translucency) of the *Lippia origanoides* formulation with 1% essential oil and 9% polysorbate 20.

The macroscopic characteristics of the formulation were a translucent liquid with a bluish reflection when exposed to light (Figure 1). After the preparation, the average droplet size was 10.87 ± 0.02 nm with a PdI of 0.131 ± 0.012.

**Figure 2:**
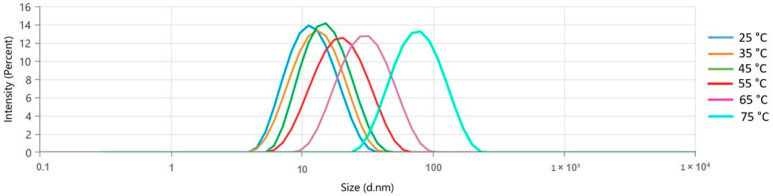
Temperature influence (25–75 °C) in the droplet size distribution by the intensity of the *Lippia origanoides* formulation.

The formulation exhibited an increase in the mean size (nm) and polydispersity index (PdI) as the temperature increased (10 °C/intervals), demonstrating a monomodal behavior at all evaluated temperatures (Figure 2). However, there was a notable increase in the average size from 25 °C (10.87 ± 0.02 nm) to 75 °C (66.09 ± 0.373). Table 2 shows each temperature’s average droplet size (nm) and PdI values. After applying heat (25 to 75 °C), the collective nanometric parameters were reassessed at room temperature (25 °C). This resulted in no discernible macroscopic alteration and nanometric values similar to those observed before the heating process (Table 3).

The increase in the mean size, PdI of the droplets, and colloidal behavior can be associated with the supersaturation of the external phase (aqueous phase) due to the increase in the solubility of the more electronegative substances (e.g., monoterpenoids and sesquiterpenoids), including the major compound (10.2%) thymol (LogP 3.28). This response to the temperature increase is characteristic of Ostwald ripening, a mass transference from smaller to higher mass droplets, a common instability mechanism of low-density colloidal systems (e.g., essential oils).

**Figure 3:**
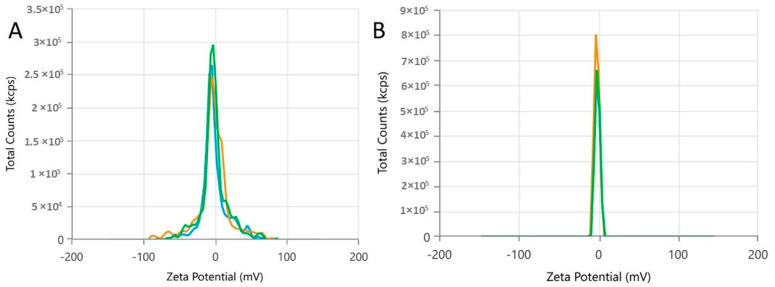
Zeta potential distribution of the *Lippia origanoides* formulation before (**A**) and after (**B**) thermal stress. The graph lines represent zeta potential measurements under different temperatures or conditions (e.g., 25 °C, 35 °C, 45 °C). Stability is indicated by the proximity or overlap of the lines.

The zeta potential (ZP) values (−1.634 ± 0.464 and −1.866 ± 0.482) indicate that the nanodroplets are negatively charged (Table 2 and Figure 3). The low magnitude of the ZP observed suggests a minor electrostatic repulsion, which can eventually lead to classical emulsion instability factors such as coalescence or aggregation. However, it is noteworthy that the occurrence of these specific colloidal destabilization events was not observed following the heating of the nanoformulation, indicating that it exhibits stability when briefly exposed to continuous temperatures.

The moderate increase in the conductivity after heating (0.280 to 0.370 mS/cm) suggests a higher free ion dissociation and presence in the external phase, possibly related to the phenol thymol (pKa 10.62) being ionized in the aqueous phase as the temperature increases. The high conductivity reflects a high ionic force that may contribute to the compression of the nanodroplet ionic layer, and, also, the presence of other ions in the external phase may partially neutralize the surface charges of the nanodroplets, thus reducing its zeta potential (mV) magnitude. Therefore, the nanoformulation stability may be more associated with surfactant (polysorbate 20) steric forces and its proportionality in the formulation than with its electrostatic repulsive forces.

### 2.3. Determination of the Acetylcholinesterase Inhibition

**Figure 4:**
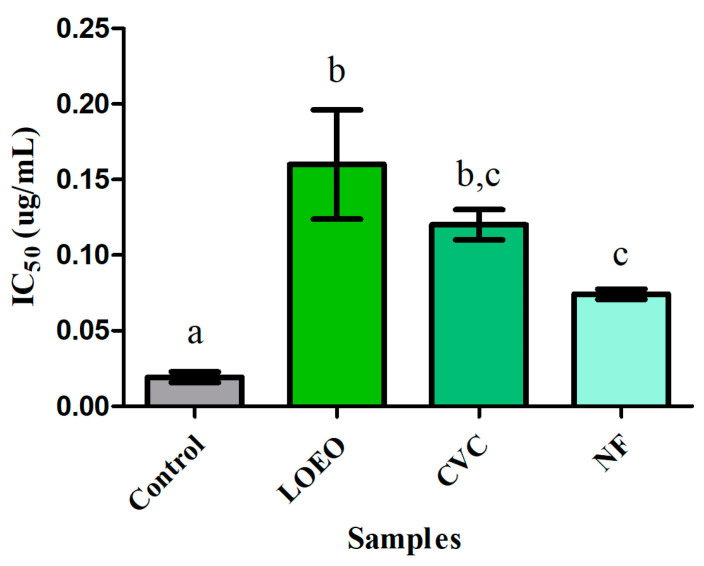
Anticholinesterase activity (IC_50_) of samples: LOEO = essential oil of *L. origanoides*; CVC = carvacrol; and NF = nanoformulation. Control = physostigmine. The data are presented as mean ± standard deviation. Statistical analysis was performed using ANOVA followed by a Tukey post hoc test, with significance defined at *p* < 0.05. The letters “a, b, c” indicate statistically different groups based on inhibitory activity against acetylcholinesterase: “a” for the control, “b” for the LOEO and carvacrol samples (similar to each other), and “c” for the nanoformulation, which showed the highest inhibitory efficacy.

The results demonstrate that all samples exhibited inhibitory activity, albeit with varying potencies compared to the control. Physostigmine, as expected, showed strong inhibitory activity, serving as a reference for the efficacy of the other samples. According to Figure 4, all samples of LOEO, carvacrol, and the nanoformulation exhibited strong inhibitory activity against acetylcholinesterase, with IC_50_ values ranging from 0.074 to 0.16 μg/mL. The LOEO showed an IC_50_ of 0.16 ± 0.036 μg/mL, while carvacrol showed an IC_50_ of 0.12 ± 0.006 μg/mL, and the nanoformulation had an IC_50_ of 0.074 ± 0.003 μg/mL. No significant difference was observed between the essential oil and carvacrol, nor between carvacrol and the nanoformulation (*p* ≤ 0.05). However, a statistically significant difference was found between the essential oil and the nanoformulation, indicating that the nanoformulation exhibited greater inhibitory activity against the acetylcholinesterase enzyme.

### 2.4. Molecular Docking

**Figure 5:**
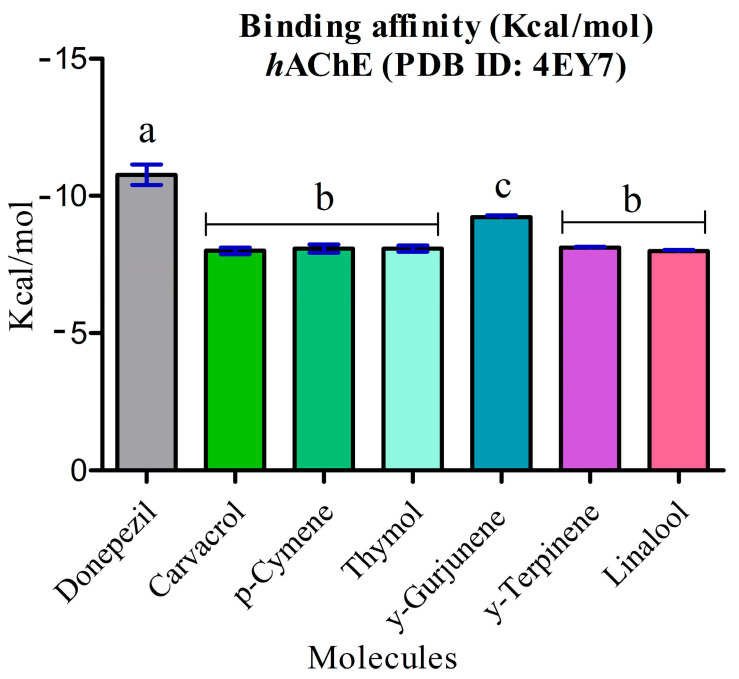
Statistical analyses of the binding energy values of donepezil compared to the major compounds of *Lippia origanoides* essential oil. Statistical analysis was performed using ANOVA followed by a Tukey post hoc test, with significance defined at *p* < 0.05. The letters “a, b, c” indicate statistically different groups based on inhibitory activity against acetylcholinesterase: “a” for the control, “b” for the LOEO and carvacrol samples (similar to each other), and “c” for the nanoformulation, which showed the highest inhibitory efficacy.

The energy calculation for all docking complexes was evaluated using the MMFF94 force field as the scoring function. The binding energy values of the major constituents of the essential oils ranged from −9.229 kcal/mol to −7.993 kcal/mol. All the major compounds of LOEO exhibited binding energy levels significantly lower than and different to (*p* ≤ 0.05) donepezil (−10.638 kcal/mol) (Figure 5). Among the compounds in the oil, γ-gurjunene demonstrated a statistically significant difference compared to the other constituents, with a binding energy of −9.229 kcal/mol.

#### 2.4.1. Analysis of the Binding Sites of Major Compounds with the AChE Enzyme

**Figure 6:**
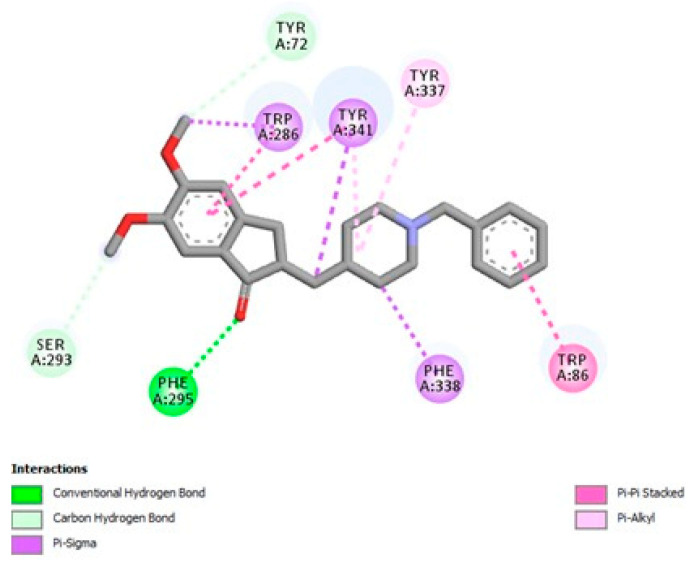
Two-dimensional diagram illustrating the interactions between donepezil and the acetylcholinesterase (AChE) enzyme (4EY7).

The initial procedure involved docking, validated by accurately re-docking the co-crystallized donepezil into the hAChE model to compare the docking results. Donepezil was docked to AChE using the same parameters and was found to have a binding affinity of −11.237 kcal/mol. Figure 6 shows the binding pocket and target residues involved in the binding interaction of donepezil.

**Figure 7:**
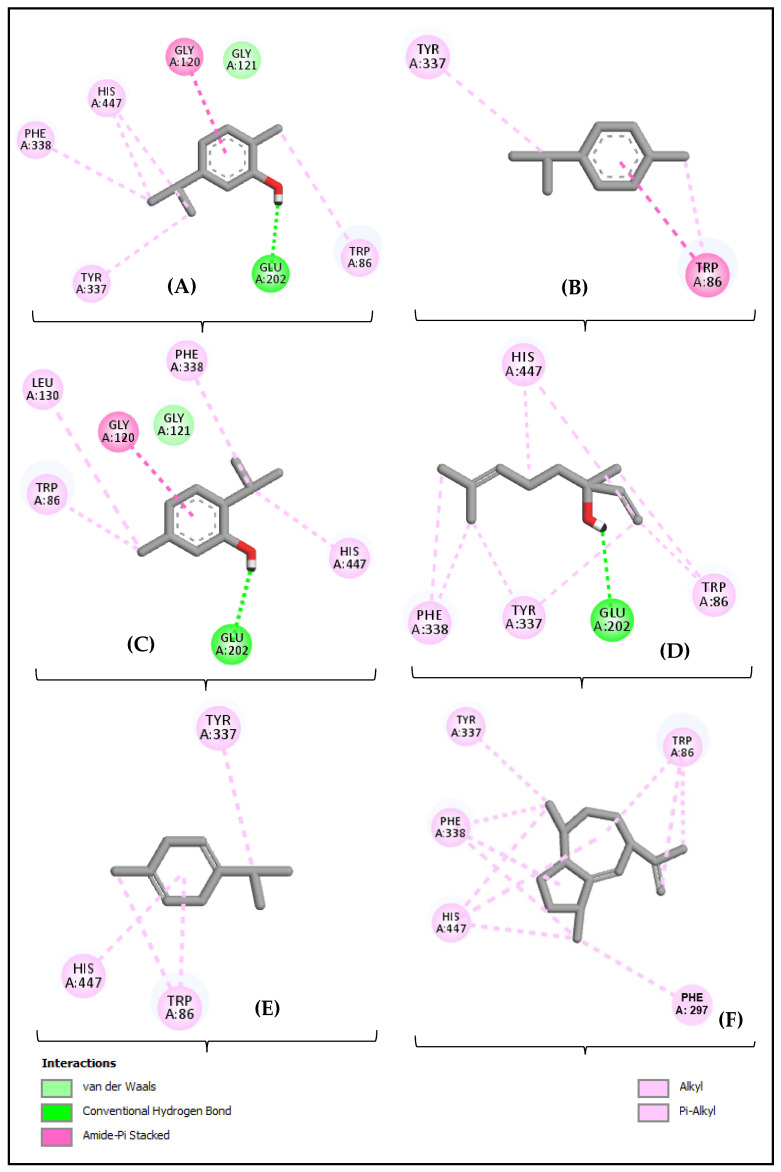
Two-dimensional diagram of the interactions between carvacrol (**A**), *p*-cymene (**B**), thymol (**C**), γ-terpinene (**D**), linalool (**E**), and γ-gurjunene (**F**), with the acetylcholinesterase (AChE) enzyme (4EY7).

Figure 7 presents the interaction diagram between the acetylcholinesterase (AChE) enzyme (4EY7) and the amino acid residues of the compounds carvacrol (Figure 7A), *p*-cymene (Figure 7B), thymol (Figure 7C), linalool (Figure 7D), γ-terpinene (Figure 7E), and γ-gurjunene (Figure 7F).

The diagrams highlight specific bonds, such as hydrogen bonds, hydrophobic interactions, and other intermolecular forces, that contribute to the stabilization of the enzyme-compound complexes. Each panel (A–F) shows how the different compounds interact with the active residues of AChE, providing insights into the mechanism of enzyme inhibition. These interactions are crucial for understanding the potential efficacy of these compounds as AChE inhibitors, which may be relevant for the development of therapeutic agents in the treatment of cholinesterase-related diseases.

#### 2.4.2. ADME Properties

Table 4 presents the in silico pharmacokinetic properties of the major constituents of LOEO, including carvacrol, *p*-cymene, thymol, γ-terpinene, linalool, and γ-gurjunene, predicted using the SwissADME and PxeADMET tools. The results indicate that all compounds exhibit high intestinal absorption (HIA = 100%) and permeability across the blood–brain barrier (BBB), suggesting good bioavailability and potential to reach the central nervous system. Additionally, the LogP values, ranging from 3.21 to 5.00, indicate that these compounds have suitable lipophilic characteristics for penetration into biological membranes. These favorable pharmacokinetic properties corroborate the results presented in Figure 4, where the compounds demonstrated anticholinesterase activity, suggesting that their inhibitory efficacy may be related to their absorption and distribution in the body. These data are essential for evaluating the therapeutic potential of these compounds in the development of pharmacological agents.

## 3. Discussion

This study aimed to explore the therapeutic potential of the essential oil of *Lippia origanoides* Kunth (LOEO), focusing on its acetylcholinesterase (AChE)-inhibitory properties. The rationale for this study is based on the growing need to develop natural and effective treatments for neurodegenerative diseases, such as Alzheimer’s, which are associated with neurotransmitter deficiency and oxidative stress. Additionally, the search for safe and sustainable therapeutic alternatives, especially those derived from plant sources, has gained prominence due to the side effects associated with synthetic drugs. LOEO, rich in phenolic compounds such as carvacrol and thymol, has shown significant potential for inhibiting AChE, a crucial enzyme in the pathogenesis of neurodegenerative diseases.

The research also investigated the feasibility of nanoformulations to improve the stability and bioavailability of these compounds, targeting pharmaceutical and clinical applications. Therefore, this study not only contributes to the understanding of the molecular mechanisms behind the biological activity of LOEO but also paves the way for the development of new therapies based on natural products, aligning with current demands for innovative and sustainable therapeutic solutions.

The analysis of the chemical composition reveals that the LOEO has a characteristic chemical profile, with carvacrol (41.2%), thymol (10.2%), *p*-cymene (11.1%), and γ-gurjunene (11.8%) as the main compounds, which is consistent with previous studies [8,17]. These phenolic compounds are known for their potent antioxidant and antimicrobial activities, highlighting LOEO as a promising source of therapeutic agents for various clinical conditions. The results also indicate a good oil yield (3.3%), reinforcing its potential for commercial applications, especially in the pharmaceutical and food industries.

In terms of biological activity, LOEO demonstrated potent acetylcholinesterase (AChE)-inhibitory activity, which is crucial in the context of treatments for neurodegenerative diseases such as Alzheimer’s. Carvacrol, being the main component, plays a central role in this effect. AChE inhibition can be attributed to its ability to form π-π interactions with key residues of the enzyme, such as TRP86 and TYR337, as evidenced by molecular interactions.

However, the therapeutic contribution of LOEO is not solely due to carvacrol. γ-Gurjunene, although present in lower concentration, has a high binding affinity for AChE, indicating its significant contribution to the observed inhibitory activity. Thymol, in turn, complements the action of these compounds, enhancing the overall effect of LOEO. Thus, the therapeutic activity of the essential oil appears to result from a synergistic effect between these compounds, making its efficacy superior to that of any isolated compound.

Compared to other *Lippia* genus species, such as *Lippia alba* and *Lippia graveolens*, similar chemical profiles emerge, dominated by carvacrol and thymol. For instance, *Lippia graveolens* oil has been reported to contain carvacrol and thymol in concentrations ranging from 30% to 60%, depending on the plant’s geographical origin and harvesting conditions [16,18]. Similarly, *Lippia alba* often exhibits a chemotype rich in citral or carvone, but studies have also identified phenolic compounds like thymol in certain variants [19]. These comparisons highlight the intra-genus variability in essential oil composition, which is influenced by genetic, environmental, and extraction factors.

The therapeutic importance of phenolic compounds such as carvacrol and thymol lies in their ability to disrupt microbial membranes, neutralize free radicals, and inhibit enzymes like acetylcholinesterase (AChE). The prominence of carvacrol in LOEO, accounting for 41.2% of its composition, is particularly noteworthy as it exceeds concentrations typically observed in other *Lippia* species and other carvacrol-rich essential oils, such as those from *Origanum vulgare* [20,21]. Additionally, the presence of γ-gurjunene, a sesquiterpene hydrocarbon, provides a unique chemical signature to LOEO, differentiating it from closely related species and potentially contributing to its bioactive profile. These findings reaffirm the value of *Lippia origanoides* as a promising source of bioactive compounds with diverse applications.

The analysis of the nanoformulation of LOEO reveals important aspects regarding its stability and physicochemical behavior under different thermal conditions, essential for its pharmaceutical and therapeutic applications. Initially, the formulation presented an average droplet size of 10.87 ± 0.02 nm and a polydispersity index (PdI) of 0.131 ± 0.012, characterizing a narrow distribution and a homogeneous formulation. PdI values below 0.2 indicate good stability and uniformity of the particles, which is crucial for controlled release and efficient absorption of bioactive compounds, such as thymol, as one of the major components of the formulation [22,23]. These parameters suggest that the formulation is suitable for therapeutic applications where particle uniformity is important in treatment efficacy.

The analysis of the temperature impact on droplet size revealed a notable increase in the average size, from 10.87 ± 0.02 nm to 66.09 ± 0.373 nm between 25 °C and 75 °C, along with a progressive increase in PdI. These results indicate that the formulation behaves in a monomodal way during thermal variation, with a tendency for droplet growth at higher temperatures. This phenomenon is a classic example of the Ostwald ripening process, where mass is transferred from smaller to larger droplets, resulting in an increased instability of emulsions. This occurs due to the increased solubility of more electronegative substances, such as monoterpenes and sesquiterpenes, present in *Lippia origanoides* essential oil [24,25].

Moreover, the behavior of the zeta potential (ZP) and conductivity of the formulation provide important insights into the electrostatic stability and compression of the ionic layer around the nanoparticles. The negative zeta potential, −1.634 ± 0.464 and −1.866 ± 0.482, suggests that the nanoparticles are slightly negatively charged, which, under ideal conditions, would help to prevent droplet coalescence. However, the low ZP magnitude indicates weak electrostatic repulsion between particles, which could favor aggregation or instability under some conditions. This phenomenon can be compensated by the presence of the surfactant (polysorbate 20), which contributes to the steric stabilization of the formulation, as recent studies on colloidal systems based on essential oils have suggested [26]. The moderate increase in conductivity from 0.280 mS/cm to 0.370 mS/cm after heating reinforces the idea that thymol, due to its high pKa, may ionize in the aqueous phase at higher temperatures, increasing ion dissociation and influencing the formulation’s stability [27].

The thermal stability observed in the *Lippia origanoides* nanoformulation, which showed no significant changes in its macroscopic characteristics after exposure to varying temperatures, is an important advantage for its use in environmental conditions that may vary, especially in pharmaceutical formulations that require stability across different temperatures ranges. The ability of the nanoparticles to maintain their characteristics even after thermal stress can be attributed to the synergistic interaction between the bioactive compounds of the essential oil and the surfactant used, which ensures the structural and functional stability of the formulation [28,29].

The combination of low polydispersity, thermal stability, and bioactive compounds such as thymol and carvacrol enhances the pharmacological potential of LOEO for therapeutic applications, such as neurological treatments or antimicrobial therapies. This research confirms the viability of the nanoformulation as a promising system for the efficient delivery of active ingredients, with a stability profile suitable for further investigation and clinical applications.

The acetylcholinesterase (AChE) inhibition assay results, presented in Figure 4, reveal a potent inhibitory activity of LOEO, carvacrol, and the nanoformulation against AChE. The IC_50_ values, ranging from 0.074 to 0.16 μg/mL, demonstrate that all tested formulations exhibit strong AChE inhibition. The nanoformulation, with the lowest IC_50_ value (0.074 ± 0.003 μg/mL), showed the highest activity, while carvacrol and *L. origanoides* essential oil exhibited similar values (0.12 ± 0.006 μg/mL and 0.16 ± 0.036 μg/mL, respectively). This finding suggests that the encapsulation of LOEO in the nanoformulation enhances its bioactivity, possibly due to an improved bioavailability and cellular uptake of the bioactive compounds, including carvacrol, which is known for its cholinesterase-inhibitory properties [30,31].

The increased inhibitory activity of the nanoformulation compared to the free essential oil and carvacrol can be attributed to the enhanced solubility and stability provided by the nanoformulation. Nanoencapsulation is known to improve the dispersibility of hydrophobic compounds and facilitate their interaction with the enzyme, leading to more efficient inhibition [32,33]. Several studies have highlighted that nanoparticles, particularly those made from surfactants like polysorbate 20, can significantly improve the pharmacokinetic properties of essential oils by protecting them from degradation and increasing their bioactivity against biological targets such as AChE [34,35]. In this study, the nanoformulation’s superior activity indicates its potential as a more effective therapeutic agent for conditions related to acetylcholinesterase dysfunction, such as Alzheimer’s disease.

Additionally, the statistical significance found between the nanoformulation and the free LOEO points to the role of nanoencapsulation in enhancing the therapeutic potential of the oil’s bioactive compounds. While carvacrol, the major compound in the essential oil, already demonstrates promising AChE inhibitory activity, the nanoformulation likely provides a more sustained and controlled release of carvacrol and other constituents, enhancing their effects. This finding aligns with recent research that shows the ability of nanoformulations to significantly improve the biological activity of essential oils, including their antioxidant and anticholinesterase properties [36,37].

In conclusion, the results underscore the importance of nanotechnology in enhancing the therapeutic efficacy of essential oils, particularly in neurological applications where acetylcholinesterase inhibition is a key therapeutic strategy. The improved AChE inhibition observed in the nanoformulation of LOEO provides compelling evidence for developing more effective and targeted treatments for neurodegenerative diseases, offering a promising avenue for future research and pharmaceutical applications.

Analyzing the binding affinity levels of the main constituents of *Lippia origanoides* essential oil (LOEO) with the AChE enzyme revealed promising data regarding its inhibitory potential. Using the MMFF94 force field as the scoring function, the binding energy values of the compounds ranged from −9.229 kcal/mol to −7.993 kcal/mol, highlighting significant interactions with the enzyme’s active site. Although all compounds showed lower binding energy values than the reference standard, donepezil (−10.638 kcal/mol), a statistically significant difference (*p* ≤ 0.05) was observed among the LOEO constituents. Among them, γ-gurjunene had the highest binding affinity (−9.229 kcal/mol), while linalool showed the lowest value (−7.993 kcal/mol). These results suggest that γ-gurjunene could play a relevant role as a natural inhibitor of AchE [38,39].

The phenolic monoterpenes carvacrol (−8.003 kcal/mol) and thymol (−8.085 kcal/mol) also exhibited competitive binding affinity levels, attributed to their ability to form π-π interactions with key residues in the enzyme’s active site, such as TRP86 and TYR337. These molecular interactions are essential for the stability of the enzyme–ligand complex, complementing evidence that phenolic compounds possess high anticholinesterase potential [40,41]. Furthermore, molecular interactions revealed that LOEO compounds formed robust hydrophobic bonds and π-σ interactions, particularly with residues such as PHE295, TYR341, and TYR72. These interaction patterns stand out for their relevance in AChE inhibition, suggesting that the structural profile of the oil’s constituents favors the formation of stable and effective complexes [42,43].

The robust molecular interaction observed between γ-gurjunene and specific residues in the active site, such as PHE295, explains its higher binding affinity. In contrast, although linalool forms bonds with catalytic residues, it does not establish high-intensity π-π interactions, justifying its lower binding affinity. These results support the hypothesis that LOEO may exert synergistic effects due to the structural diversity of its constituents. For example, carvacrol and thymol, with their strong π-π and π-σ interactions, may enhance the overall inhibitory effect of the oil when combined with other constituents, such as γ-terpinene and *p*-cymene, which exhibit moderate hydrophobic interactions and hydrogen bonding [44,45].

The association between the binding affinity data and molecular interactions strengthens the understanding of LOEO’s anticholinesterase potential. While γ-gurjunene stands out in affinity, carvacrol and thymol provide additional advantages due to their diversified interactions, particularly with critical residues such as TYR341, TRP86, and PHE33. This synergistic profile suggests that LOEO as a whole could be a promising natural source of AChE inhibitors, combining significant affinity with effective molecular interactions. These factors are crucial for the development of natural therapies against neurodegenerative diseases, such as Alzheimer’s disease [46,47].

Additionally, the comparison with the standard inhibitor donepezil, which showed the highest binding affinity (−10.638 kcal/mol), reinforces the relative efficacy of LOEO compounds. Although none of the isolated constituents reached the same level of affinity, the combined interaction profile and binding affinity of LOEO constituents indicate a synergistic mechanism of enzymatic inhibition. This synergistic approach could be a significant advantage in terms of safety and therapeutic efficacy, especially when compared to synthetic drugs, which often have undesirable side effects [48,49].

Besides acetylcholinesterase inhibition, carvacrol and thymol demonstrated additional neuroprotective properties that may contribute to the treatment of neurodegenerative diseases. Previous studies indicate that carvacrol has significant antioxidant activity, neutralizing free radicals and reducing oxidative stress, a key factor in the progression of diseases like Alzheimer’s [8]. Additionally, carvacrol exhibits anti-inflammatory effects by inhibiting the release of pro-inflammatory cytokines and modulating signaling pathways such as NF-κB, which may help to reduce neuroinflammation associated with neuronal damage [50]. Thymol, in turn, also has antioxidant and anti-inflammatory properties, protecting neuronal cells from oxidative damage and apoptosis. These combined effects suggest that LOEO, rich in carvacrol and thymol, may act in a multifaceted manner in protecting the central nervous system, not only by inhibiting AChE but also by mitigating cellular damage caused by oxidative stress and inflammation. These neuroprotective properties enhance the potential of LOEO as a promising therapeutic approach for neurodegenerative diseases.

The therapeutic activity of LOEO appears to result from a synergistic effect between its main compounds, such as carvacrol, thymol, and γ-gurjunene. Although carvacrol, as the major component, plays a central role in acetylcholinesterase (AChE)-inhibitory activity, γ-gurjunene, with its high binding affinity to the enzyme, also makes a significant contribution. Thymol, in turn, complements the action of these compounds, enhancing the overall effect of LOEO. Therefore, the therapeutic effect of the essential oil cannot be attributed to a single compound but rather to the synergistic interaction between them, resulting in a more robust activity than that observed with any isolated compound.

These results, combined with the thermal and structural stability already discussed in other parts of this study, reinforce the potential of LOEO and its constituents as multifunctional agents for AChE inhibition. The ability to form stable, high-affinity complexes with the enzyme underlines its relevance as a natural alternative for the development of phytotherapeutic treatments against neurodegenerative diseases, such as Alzheimer’s disease [51,52].

The absorption parameters evaluated, such as LogP, PCaco-2, HIA (%), and PMDCK, indicate a favorable profile for the oral bioavailability of the major constituents of *Lippia origanoides* essential oil (LOEO). The LogP values, reflecting lipophilicity, ranged from 3.21 (linalool) to 5 (γ-gurjunene). LogP values close to 5 are generally considered the upper limit for good absorption, indicating that γ-gurjunene exhibits the highest lipophilicity among the compounds. This may facilitate its passage through lipid membranes but suggests a higher risk of low aqueous solubility [53,54]. On the other hand, compounds such as linalool (3.21) and carvacrol (3.81) demonstrate a more favorable balance between lipophilicity and aqueous solubility, aligning with desirable properties for effective drugs [53].

The PCaco-2 parameter, a predictor of intestinal permeability, indicated high values for carvacrol (38.0121), thymol (38.0122), and linalool (29.355), while γ-terpinene (23.6401), γ-gurjunene (23.6411), and *p*-cymene (23.4337) showed lower values. These results suggest that carvacrol and thymol have a greater ability to cross the intestinal barrier than the other constituents, which can directly influence the efficacy of the compounds when administered orally [50,51]. All compounds exhibited 100% human intestinal absorption (HIA), corroborating their excellent oral bioavailability. Regarding the PMDCK parameter, which assesses permeability in renal cell cultures, γ-terpinene (244.909) and *p*-cymene (237.507) showed the highest values, indicating greater potential for absorption through alternative pathways [55].

For the distribution parameters, all compounds demonstrated high plasma protein binding (LPP), with values of 100%, indicating that most of these compounds would be bound to plasma proteins, reducing their free fraction for pharmacological activity. However, this can also act as a reservoir, prolonging the half-life of the compounds in the body [56,57].

The blood–brain barrier penetration coefficient (BBB) revealed varying values among the compounds, with γ-gurjunene showing the highest value (13.4717), followed by γ-terpinene (8.03745). These results highlight γ-gurjunene’s potential to efficiently cross the blood–brain barrier, which is highly relevant for neuroprotection and acetylcholinesterase (AChE) inhibition applications. On the other hand, *p*-cymene (4.96983) and linalool (6.12506) exhibited lower BBB penetration, which could be advantageous in applications where activity should be limited to the peripheral system [55,56].

Overall, the in silico results suggest that the pharmacokinetic profile of LOEO constituents aligns with their previously observed bioactive properties, such as AChE inhibition. Compounds with higher absorption and distribution potential, such as γ-gurjunene, γ-terpinene, carvacrol, and thymol, may significantly contribute to the essential oil’s efficacy in therapeutic contexts. Particularly, γ-gurjunene’s ability to cross the BBB suggests a central role in LOEO’s neuroprotective effect, while other compounds like carvacrol and thymol may act complementarily, providing structural stability and synergistic interactions with target receptors [56,57].

These results emphasize the need for additional experimental studies to confirm the synergistic effects and bioavailability of the compounds in the body. Moreover, strategies such as nanoencapsulation can be employed to optimize the delivery of bioactive compounds, particularly those with high lipophilic properties, such as γ-gurjunene, ensuring their efficacy in therapeutic applications [58].

The main strengths of this study include the comprehensive analysis of LOEO through a combination of chemical, biological, and pharmacokinetic techniques, which provide a detailed understanding of the therapeutic properties of LOEO and its nanoformulations. The use of nanoencapsulated formulations demonstrated not only the stability of the essential oil but also its increased efficiency in inhibiting AChE, highlighting its therapeutic potential in neurodegenerative diseases.

Additionally, the molecular modeling results provided precise insights into the interaction mechanisms of the key compounds with the enzymatic target. However, some limitations should be noted. Firstly, experimental data were primarily obtained in vitro, and clinical validation is essential to confirm the therapeutic efficacy of LOEO in humans. Furthermore, although the bioavailability results indicate that the compounds of LOEO have a good pharmacokinetic profile, absorption and distribution in the body need to be better understood through experimental studies. Future work should delve deeper into these issues and explore the applicability of nanoformulations in animal models.

## 4. Materials and Methods

### 4.1. Plant Material and Obtaining the Samples

The aerial parts (leaves and thin branches) of *L. origanoides* Kunth were collected in the village of Alter do Chão (2°30′31.0″ S and 54°57′00.0″ W), Santarém, Pará, Brazil, in May and July 2021. Vouchers were prepared and deposited in the herbarium of the University of Juiz de Fora, Minas Gerais, under the number CESJ-64029. This research was registered in SisGen (Sistema Nacional de Gestão do. Patrimônio Genético e do Conhecimento Tradicional Associado) under the number A965D42.

The plant material (leaves and thin branches) was dehydrated at room temperature for 7 days, and then the leaves were removed from the branches, manually crushed, and subjected to the essential oil extraction process. The essential oils from both samples were obtained by hydrodistillation using a Clevenger-type apparatus for 120 min. The obtained essential oils were subjected to centrifugation with anhydrous sodium sulfate to remove water, and the yield was calculated based on the weight of the material used [59]. The extraction resulted in an average yield of 3.3 mL of essential oil per 100 g of plant material. The oil samples were placed in amber glass bottles, hermetically sealed, and stored at 5 °C for subsequent volatile constituents and biological assay analysis. Additionally, the isolated compound carvacrol was obtained commercially from Sigma-Aldrich^®^ (St. Louis, MO, USA, batch: SLBZ8573) with a purity of 99.99%.

### 4.2. Analysis of the Volatile Constituents of the Samples

The essential oil was analyzed by a gas chromatography system coupled to a mass spectrometer (GCMS-QP2010 Ultra, Shimadzu Corporation, Tokyo, Japan), equipped with an auto injector (AOC-20i—Shimadzu Corporation, Tokyo, Japan) and CGMSsolution software (Version 4.20), which contains spectra libraries [60], including FFNSC 2 [61], following the method described by us [62]. A fused silica capillary column (Rxi-5ms, Restek Corporation, Bellefonte, PA, USA) of 30 m × 0.25 mm (diameter) × 0.25 µm (film thickness), coated with 5% diphenyl dimethylpolysiloxane, was used as a stationary phase. The analysis conditions were as follows: helium drag gas (99.995%); split ratio mode at 1:20; injection of 1 µL of the sample (3 µL of the essential oil in 500 µL of hexane); ionization energy by electronic impact (EI) 70 eV; injector temperature: 250 °C; oven temperature: initially set at 60 °C, then increased to 240 °C at a rate of 3 °C/min, and held for 10 min; ion source temperature: 200 °C; transfer line temperature: 250 °C.

Quantitative data on the volatile constituents were obtained via peak area normalization using a gas chromatograph (GC 6890 Plus series, Agilent, Santa Clara, CA, USA) coupled to a flame ionization detector (FID), which was operated under similar conditions to the GC-MS system. The mass spectra were obtained by automatic scanning at 0.3 scans/second, with mass fragments of 35–400 *m*/*z*. The compounds found in the ion chromatograms were identified by comparing the mass spectra (molecular mass and fragmentation pattern) with those found in the system’s CGMSsolution library and by comparison with the retention indexes. The linear equation of Van den Dool and Kratz (1963) [63] was used to calculate the retention index (RI) for each volatile component, with the use of a standard homologous series of C8–C40 n-alkanes (Sigma-Aldrich, St. Louis, MO, USA), following the equation displayed below.

$$RI=\left(\frac{100}{{TR}_{\left(HA\right)}-{RT}_{\left(HB\right)}}\right)\times {RT}_{\left(X\right)}-{TR}_{\left(HB\right)}+{IK}_{\left(HB\right)}$$

where ${IK}_{\left(HB\right)}$ = the Kóvats index of hydrocarbon that elutes before the component; ${TR}_{\left(HA\right)}$ = the retention time of hydrocarbon that elutes after the component; ${TR}_{\left(HB\right)}$ = the retention time of hydrocarbon that elutes before the component; ${RT}_{\left(X\right)}$ = the retention time of the essential oil component.

### 4.3. Preparation, Characterization, and Thermal Stress of the Lippia origanoides Formulation

The nanoemulsification was realized by the low-energy phase inversion method [64]. The oil phase was composed of 1% (*w*/*w*) of LOEO and 9% (*w*/*w*) of polysorbate 20 (Sigma-Aldrich, St. Louis, MO, USA), while the aqueous phase was composed of 90% (*w*/*w*) of deionized water. The formulation was prepared by homogenizing the oil phase in a vortex homogenizer (AP56, Pheonix Luferco, Araraquara, SP, Brazil) for 2 min; then, the aqueous phase was slowly dripped into the oil phase under constant homogenization. The resultant formulation was characterized in a Zetasizer Advance Lab Blue (Malvern Panalytical, Malvern, UK) by its average droplet size (nm) and polydispersity index (PdI) by dynamic light scattering (DLS) and zeta potential (ZP) by electrophoretic light scattering (ELS). The refractive index of LEOEO utilized was 1.52, with a glass cuvette (PCS8501, Malvern Panalytical, Malvern, UK), and a DLS 90° detection angle of detection. The analysis was realized in triplicate without diluting the formulation.

The colloidal behavior and nanometric parameters, including droplet size and polydispersity index (PdI), of the *Lippia origanoides* formulation were systematically evaluated across a temperature range of 25 to 75 °C, with analyses conducted at 10 °C intervals, and a pos-heated formulation at 25 °C to evaluate its thermal stability [65]. The zeta potential (ZP) and conductivity (mS/cm) were evaluated before and after the temperature increase.

### 4.4. Determination of the Cholinesterase Inhibition

For the in vitro anticholinesterase assays, the enzyme acetylcholinesterase type VI-S, obtained from *Electrophorus electricus* (lyophilized powder, C3389-2Ku, Sigma-Aldrich, St. Louis, MO, USA, batch: SLBZ8573), was used. The eserine (physostigmine) diluted in methanol was used as standard anticholinesterase inhibitor (Sigma-Aldrich, batch: BCBC4171V). A standard curve was used to define the concentration used in the tests.

#### Quantitative Assay

The assay for the quantification of acetylcholinesterase inhibition of the samples (essential oil, nanoformulation, and isolated carvacrol) was adapted from Ellman’s method [66], with modifications as described by [67]. In summary, three buffers were produced for the quantitative test, denominated as A, B, and C. These being the following: buffer A = 50 mM Tris/HCl, pH 8, dissolved in ultrapure water; buffer B = 0.1% bovine serum albumin in buffer A; and buffer C = 0.1 M NaCl and 0.02 M MgCl_2_·6H_2_O dissolved in buffer A.

In a total volume of 1 mL, 415 µL of buffer A, 10 µL of the samples (diluted in methanol, buffer, and Tween 80) at different concentrations (100, 50, 25, 12.5, 6.25, and 3.12 ug/mL), and 75 µL of acetylcholinesterase enzyme, containing 0.2 U/mL, were added. The samples were then incubated for 15 min at 25 °C. After incubation, 75 µL of a solution of 1.83 mM AChI (acetylthiocholine iodide) (Sigma-Aldrich, Steinheim, Germany) and 425 µL of 3 mM DTNB (5,5′-dithiobis[2-nitrobenzoic acid]) (Sigma-Aldrich, Steinheim, Germany) were added and the mixture was incubated for 30 min at 25 °C under a light source. The absorbance of the mixture was measured at 412 nm in a UV spectrophotometer (NOVA, 3300). Physostigmine was used as the standard drug, and a dilution solution was used as negative control (buffer A, methanol, and Tween 80 at a ratio of 2:2:1). The percentage of inhibition of enzyme activity was calculated according to the equation % = [(A_0_ − A_1_)/A_0_] ×100, where A_0_ is the absorbance of the control without the essential oil and A1 is the absorbance of the essential oil sample at different concentrations. All tests were performed in triplicate. The sample concentration that provided 50% inhibition (IC_50_) was obtained by constructing graphs of the percentages of inhibition versus the concentration of the inhibitor. The non-linear regression parameters for the curve were plotted, and the IC_50_ values were obtained using the Microsoft Excel 2019 software.

### 4.5. Molecular Modeling and ADME Properties

#### 4.5.1. Preparation of the Ligands

The chemical structures of the major compounds in the essential oil of *L. origanoides* were obtained in “.mol” format from the National Institute of Standards and Technology (NIST) database (available at https://www.nist.gov/, accessed on 14 November 2024). Subsequently, using ChemSketch software (version 2021.2, ACD/Labs, Toronto, ON, Canada), the molecules had their 3D structures optimized by molecular mechanics methods.

#### 4.5.2. Preparation of the Crystallographic Structure of hAChE (PDB: 4EY7)

To perform the molecular docking simulation, the crystallographic structure of human acetylcholinesterase (hAChE) was obtained from the Protein Data Bank (PDB) (https://www.rcsb.org/, accessed on 14 November 2024) under ID code 4EY7. Subsequently, BIOVIA Discovery Studio^®^ v. 20.1.0 was used to process the crystallographic complex. Thus, the co-crystallized ligand, enzymatic cofactors, and water molecules were removed, and the xyz coordinates of the donepezil binding site were determined.

#### 4.5.3. Re-Docking

The approved drug, donepezil, used here as a positive control, was docked to the protein binding site (PDB: 4EY7) via the DockThor web server (https://www.dockthor.lncc.br/v2/, accessed on 14 November 2024). Molecular docking was performed using a genetic algorithm with multiple solutions and the Merck Molecular Force Field 94 (MMFF94) as the scoring function. All of donepezil’s rotatable bonds were considered at this stage, and the pre-selected precision settings of the algorithm, as well as the grid box size (20 × 20 × 20), were maintained.

The RMSD (root mean square deviation) value was calculated using Discovery Studio software (version 2021, BIOVIA, San Diego, CA, USA). Thus, the molecular docking parameterization was established based on an RMSD value below 2.0 Å, indicating that the docking protocol can be used for docking other ligands.

#### 4.5.4. ADME Properties

The selected compounds were subjected to pharmacokinetic analysis using the online prediction tool Swiss ADME (available at http://www.swissadme.ch/, accessed on 14 November 2024). This program provides physicochemical properties and ADME (absorption, distribution, metabolism, and excretion) parameters associated with the pharmacokinetics of each compound. Calculated data included lipophilicity values (LogPo/w) and blood–brain barrier (BBB) permeability. Additional properties such as plasma protein binding (PPB), permeability in MDCK and CACO-2 cells, and human gastrointestinal absorption (HIA) were assessed using the PreADMET server (https://preadmet.webservice.bmdrc.org/adme/, accessed on 14 November 2024).

#### 4.5.5. Statistical Analysis

Data from the quantitative anticholinesterase assay were analyzed using the Prism 5 software with one-way ANOVA, followed by the Tukey test with multiple comparisons, at a significance level of *p* < 0.05. The GraphPad Prism 8.0.1 software was used for the statistical evaluation of binding affinity values through a one-way ANOVA, which was followed by Sidak’s multiple comparison test to evaluate and infer differences to the chosen pivot molecule.

## 5. Conclusions

This study confirms the potential of *Lippia origanoides* essential oil (LOEO) as a promising source of bioactive compounds with significant implications for pharmaceutical and neurological research. The chemical composition of LOEO, including carvacrol, thymol, and γ-gurjunene, along with its potent acetylcholinesterase (AChE)-inhibitory activity, positions LOEO as a valuable candidate for therapies targeting neurodegenerative diseases, particularly Alzheimer’s disease.

The results show that the LOEO nanoformulation offers additional benefits, such as an enhanced bioactivity and stability of the bioactive compounds, highlighting the advantages of nanotechnology in improving bioavailability and treatment efficacy. Molecular docking analysis supports LOEO’s AChE-inhibitory potential, demonstrating strong binding affinities, while in silico pharmacokinetics indicate favorable absorption and good blood–brain barrier penetration.

Although the results are promising, this study acknowledges limitations, such as the lack of in vivo validation and the stability assessment of the nanoformulation. Future studies should prioritize in vivo pharmacodynamic investigations, evaluations in animal models, long-term stability testing, and the analysis of other pathways related to neurodegenerative diseases.

## Figures and Tables

**Table 1:** Volatile constituents of *Lippia origanoides* essential oil.

Constituents	IR_calc_	IR_lit_	Conc. (%)
α-Pinene	932	932	0.16
Myrcene	989	988	1.84
α-Terpinene	1016	1014	1.09
*p*-Cymene	1023	1020	11.1
1,8-Cineole	1029	1026	1.07
γ-Terpinene	1057	1054	3.49
Linalool	1099	1095	4.60
α-Terpineol	1190	1186	0.13
Thymol Methyl Ether	1233	1232	1.56
Safrole	1287	1285	1.98
Thymol	1292	1289	10.20
Carvacrol	1305	1298	41.20
(*E*)-Caryophyllene	1419	1417	2.78
α-Humulene	1453	1452	0.25
γ-Gurjunene	1494	1495	11.8
Caryophyllene Oxide	1582	1582	1.09
1-*epi*-Cubenol	1630	1627	0.28
Monoterpene hydrocarbons			18.75
Oxygenated monoterpenes			57.59
Sesquiterpene hydrocarbons			16.81
Oxygenated sesquiterpenes			1.37
Total (%)			95.00

RI_calc_ = calculated retention time; RI_lit_ = retention time claimed in the literature [16]. Conc. (%) = percentage concentration of the constituents.

**Table 2:** Average droplet size and polydispersity index of the *Lippia origanoides* formulation at rising temperatures (25–75 °C).

Temperature (°C)	Average Droplet Size (nm)	Polydispersity Index
25	10.87 ± 0.020	0.131 ± 0.012
35	12.02 ± 0.097	0.135 ± 0.013
45	14.10 ± 0.040	0.136 ± 0.011
55	17.79 ± 0.155	0.155 ± 0.010
65	26.83 ± 0.138	0.173 ± 0.005
75	66.09 ± 0.373	0.193 ± 0.005

The data are presented as mean ± standard deviation (*n* = 3).

**Table 3:** Macroscopic characteristics, average size, polydispersity index, zeta potential, and conductivity before and after thermal stress (25 °C).

Thermal Stress	Macroscopic Characteristics	Average Size (nm)	Polydispersity Index	Zeta Potential (mV)	Conductivity (mS/cm)
Before	Translucent	10.87 ± 0.020	0.131 ± 0.012	−1.634 ± 0.464	0.2809
After	Translucent	10.16 ± 0.050	0.069 ± 0.011	−1.866 ± 0.482	0.3709

The data are presented as mean ± standard deviation (*n* = 3).

**Table 4:** In silico pharmacokinetic prediction of the major constituents of *Lippia origanoides* essential oil.

Molecule	Absorption				Distribution	
	LogP [a]	P_Caco−2_ [b]	HIA (%) [b]	P_MDCK_ [b]	LPP (%) [b]	BHE [a]
Carvacrol	3.81	38.0121	100	87.3307	100	6.38799
*p*-Cymene	3.90	23.4337	100	237.5070	100	4.96983
Thymol	3.34	38.0122	100	87.3307	100	6.38802
γ-Terpinene	3.36	23.6401	100	244.9090	100	8.03745
Linalool	3.21	29.3550	100	115.4210	100	6.12506
γ-Gurjunene	5.00	23.6411	100	57.0682	100	13.4717

[a] Analysis conducted on the SwissADME web server (http://www.swissadme.ch/index.php#, accessed on 14 November 2024); [b] analysis conducted on the PreADMET web server (https://preadmet.webservice.bmdrc.org/adme/, accessed on 14 November 2024).

## Data Availability

The data associated with this study have not been deposited in a publicly available repository; however, they can be provided upon request.

## Supplementary Materials

The following supporting information can be downloaded at: https://www.mdpi.com/article/10.3390/molecules30071554/s1.
