# Supplementary figures and images for: Essential Oil of Lippia origanoides Kunth: Nanoformulation, Anticholinesterase Activity, and Molecular Docking
Source: Molecules. 2025 Mar 31;30(7):1554. doi: 10.3390/molecules30071554 (PMC11990080; doi:10.3390/molecules30071554)

# Supplementary Material

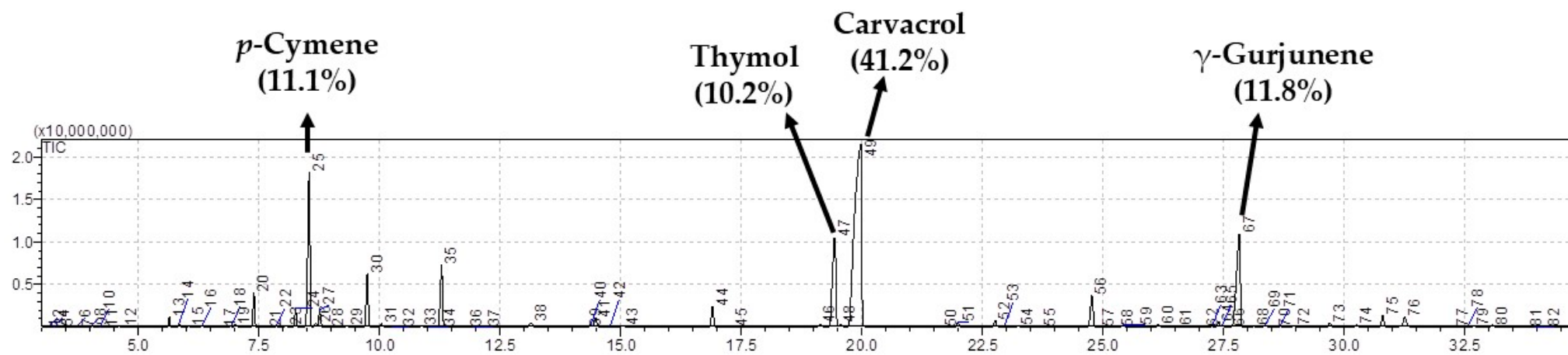

Supplement: Supplementary file 1 [file molecules-30-01554-s001.zip › molecules-3471133-supplementary.pdf]
